# Non-medical cannabis use: international policies and outcomes overview. An outline for Portugal

**DOI:** 10.47626/2237-6089-2021-0239

**Published:** 2021-04-26

**Authors:** Pedro Cabral Barata, Filipa Ferreira, Catarina Oliveira

**Affiliations:** 1 Regionspsykiatrien Vest Herning Denmark Regionspsykiatrien Vest, Herning, Denmark.; 2 Departamento de Psiquiatria e Saúde Mental Hospital Prof. Dr. Fernando Fonseca Amadora Portugal Departamento de Psiquiatria e Saúde Mental, Hospital Prof. Dr. Fernando Fonseca, Amadora, Portugal.; 3 Departamento de Psiquiatria e Saúde Mental Unidade Local de Saúde de Castelo Branco Castelo Branco Portugal Departamento de Psiquiatria e Saúde Mental, Unidade Local de Saúde de Castelo Branco, Castelo Branco, Portugal.

**Keywords:** Cannabis legalization, non-medical cannabis, Portugal, cannabis decriminalization

## Abstract

**Introduction:**

Cannabis is probably the most commonly used illicit drug. It is often regarded as a relatively nonharmful experience, even though evidence indicates otherwise. Legalization of non-medical cannabis, which has already taken place in several countries, is currently a controversial issue.

**Objective:**

To provide an up-to-date overview of current models and policies and their outcomes that can inform future political decisions regarding non-medical cannabis use.

**Methods:**

PubMed/MEDLINE and Google Scholar scientific databases were searched for articles written in English, Spanish, and Portuguese published between 1990 and December 2020. The reference lists of these articles were similarly used as bibliography sources. Gray literature was also included.

**Results:**

While non-medical cannabis has been decriminalized in many countries, it has only been legalized in Uruguay, Canada, and some U.S. states. Several benefits of legalization were identified: decreases in cannabis-related crimes, law-enforcement and judicial costs; reduction in synthetic cannabis supply; decline in black economies and possible diminution of other illegal drug buying; and tax revenue increases. Reported legalization problems included: increases in cannabis use; cannabis-related disorders; and cannabis-related accidents and hospitalizations. Harm-reduction strategies are available in the scientific literature.

**Conclusion:**

Growing, although incomplete, evidence exists to guide policy makers, minimize cannabis-related harm, and positively contribute to public health, if the legalization path is to be followed. Dialogue between legislators and science should be encouraged. There are more than a few legalization pathways, with diverse economic, social and health wellbeing effects. Public health-driven, instead of profit-driven models, seem to offer the most benefits regarding non-medical cannabis legalization. Most of the true public health effects of cannabis legalization are still unknown, for we are still in the early stages of these policies and their implications. Future studies should address the medium-to-long-term social, economic, and health consequences of legalization policies.

## Introduction

Cannabis is possibly the most widely-used illicit drug in the world.^1^ Non-medical (commonly referred as “recreational”) cannabis consumption is often regarded as a relatively non-harmful experience.^2^ However, the risks of acute and chronic health impairments associated with cannabis use are well documented,^3^ particularly mental health harm.^4^ Its long-term use has also been associated with a host of deleterious mental health, developmental, and psychosocial outcomes.^5^ Although it is difficult to establish a direct causal link between cannabis use and psychotic disorders, prospective, longitudinal, and epidemiological studies consistently report an association between cannabis usage and schizophrenia in which drug use precedes psychosis, controlling for other risks factors.^6-9^ Not only may cannabis use play a role in the first psychotic episode, but its continued consumption is also associated with poorer prognosis and with increased relapse rates, even when controlling for other factors.^9,10^ Particularly when used heavily (defined as “DSM-IV cannabis use disorder” or “at least weekly cannabis use”), cannabis may be associated with an increased risk of developing depressive disorders.^11^ Adolescent cannabis use has been linked to increased risk of developing depression and suicidal behavior later in life, even in the absence of a premorbid condition.^12^ Prenatal exposure to cannabis is also a matter of concern, as associations have been found with higher offspring psychopathology during middle childhood, sleep problems, lower cognition, lower gray and white matter volumes, lower total intracranial volume, and reduced birth weight,^13^ as well as affective symptoms and ADHD.^14^ A recent meta-analysis showed an association between cannabis use and violence when considering individuals with severe mental illnesses^3^ and lifetime combustible cannabis use was associated with a 2.12 hazard ratio of developing tracheal, bronchus, or lung cancer over nonusers.^15^ The acute intoxicating effects of use have been associated with a higher risk of motor vehicle collision^16-18^ and marginally associated with severe and fatal injuries.^5^

Recently, several countries have been discussing the possibility of legalizing largescale commercial cannabis production and sale of cannabis for non-medical use, sometimes called recreational use, after others had already established decriminalization and depenalization policies.

Non-medical cannabis legalization is a controversial issue due to various moral, ethical, public health, legislative, and logistic issues associated with the matter.^19^ Decriminalization differs from legalization in the sense that purchase, possession, and consumption of the drugs in question remain criminal offenses and are subject to criminal actions (e.g., small legal fines), although usually falling short of imprisonment.^20-22^ Legalization, on the other hand, means that cannabis would be legally available for adults and allows governments to regulate its use and sale.^20-23^

This review aims to provide an overview of current models and their outcomes that may help to inform future non-medical cannabis use policies. Taking the *status quo* of Portuguese cannabis usage and legislation as a point of departure, we comprehensively reviewed the existing literature regarding the experiences of countries where this drug has been decriminalized and, chiefly, legalized for non-medical use.

## Methods

We performed an up-to-date and comprehensive review based on scientific article searches and use of gray literature.

Article searches were conducted on the PubMed/MEDLINE database (https://www.ncbi.nlm.nih.gov/pubmed/) and Google Scholar (https://scholar.google.com/). Three different strategies of combinations of keywords were used: “Cannabis recreational use legalization” OR “non-medical cannabis legalization”; “Cannabis,” “cannabis use,” “marijuana,” “cannabis legalization,” “cannabis recreational use,” “non-medical cannabis” AND “Netherlands,” “Uruguay,” “USA,” “Canada,” “Spain,” “Portugal”; and “Portugal” AND “drug decriminalization law” OR “Portuguese drug law.”

Titles and abstracts were screened for inclusion according to their relevance to the paper’s objectives. These articles’ reference lists were also used as bibliographic sources. We included peer-reviewed articles written in English, Spanish, and Portuguese, published between 1990 and December 2020. We included articles related to cannabis legalization policies across the globe and to the Portuguese drug decriminalization laws.

The gray literature consisted of governmental and national legal documents, governmental and non-governmental organizations research reports, addiction textbooks, newspaper articles related to the legalization of cannabis, and Portuguese drug laws. We included gray literature written in English, Spanish and Portuguese. This literature was included because of the fact that such documents can hold essential and up-to-date information unavailable solely through article databases searches. While being aware of the risk of incorporating low-quality research, as well as possible bias due to political and social agendas, we aimed to include up-to-date gray literature of high quality and from reliable sources, attempting to cross-check the information whenever possible.

## Portuguese legislation and cannabis use data

### Legislation and outcomes

In Portugal, cannabis is considered an illicit drug. Illicit drug possession and use were decriminalized in Portugal in 2001 (Lei nº 30/2000 de 29 de Novembro; Decreto-Lei nº 130-A/2001, de 23 de Abril),^20,24-26^ as addictive behaviors were starting to be seen as a consequence of a health disorder and legislators began to acknowledge that criminalization would not help reduce drug use.^24^ The initial objective of such legislative changes was to focus on primary prevention for substance addicted individuals by introducing them to health care services,^25,26^ while developing specialized substance addiction help and support.^24,25^ This policy aimed to reduce harm, stop pointless punishment, and achieve better control over the drug problem.^21,24-26^ The amount of each drug that a person can possess before being treated as a drug dealer is specified in the drug law from 2000.^21,24^ It is generally thought to be the quantity one person would consume in 10 days. For cannabis this is 25 grams and for hashish it is 5 grams.^20,21,25^ If caught by the police with such small doses, individuals are issued with a notice requiring them to attend a Dissuasion Commission within 72 hours.^20,25^ The Dissuasion Commission is oriented towards a comprehensive assessment of each person’s situation and the action to be taken (e.g., banning them from practicing certain professions; imposing fines; requiring periodic attendance at a designated place).^20,25^

While the data on the outcomes of the Portuguese drug reform of 2001 is complex, it appears to have been positive in the 10 years that followed it, as consistently found across the literature:

- Declining trends for drug-related morbidity (e.g., reduction in heroin and cocaine-related seizures; decrease in the incidence of substance addicted individuals among newly infected human immunodeficiency virus [HIV] patients)^27-30^;
- Decline of drug-related mortality up to 2013,^27-29^ with an upward trend since 2014 – possibly partially explained by the implementation of a national online mortality registration system that improved compilation of all deaths in the country^29^;
- Lower lifetime prevalence rates for almost every category of drug use, in the post-decriminalization period and for several age groups up to 2008^27^;
- Reduction in the number of drug-related offenses up to 2011^28,29^ with an upward trend thereafter.^29^ The upward tendency is mainly correlated with trafficking (71%) and is mostly due to cannabis and ecstasy, as drug-related offenses involving cocaine and heroin have both been declining since 2002^31^;
- Overall decrease of patients entering treatment for heroin dependence,^32^ with possible links to falls in the number of newly dependent individuals^32^ and governmental budget constraints following the general elections that led to a change in the ruling party^28^;
- Reduction of drug-related judicial costs.^28,30^

Even though drug use is not legal in Portugal – but a social offence^20,24,25^ – the legalization of cannabis cultivation, sale and use has, in the last 4-5 years, become a recurrent issue in Portuguese society, largely due to the unsatisfying results obtained under the prohibitionist pathway. This matter has already been reflected in Portuguese research, as a recent paper by Baptista-Leite et al.^24^ regarding the topic of cannabis legalization in Portugal has argued that such a debate should be encouraged, basing its argument on a public health perspective.^24^

### Cannabis use data

The estimated prevalence of cannabis use at any period of life in the Portuguese population is 9.7%, lower than the average European prevalence (15.1%).^33^ Nonetheless, the prevalence rates of both last 12-month and lifetime cannabis consumption have been rising, since at least 2012.^31^ Moreover, it is estimated that Portugal has a higher prevalence of moderate/heavy consumers, when compared with the average European prevalence (2.3-3.2 vs. 1%).^33,34^

A recent retrospective observational study that analyzed all hospitalizations that occurred in Portuguese public hospitals from 2000 to 2015, reported a total of 3,233 hospitalizations with a primary diagnosis of psychotic disorder or schizophrenia and with a secondary diagnosis of cannabis abuse or dependence.^35^ The authors described a 29.4 times increase in the number of hospitalizations for psychotic disorders or schizophrenia that were associated with cannabis use over the study period, with the secondary diagnosis of cannabis use growing from 0.87 to 10.60%.^35^ The authors hypothesized that the rise in hospitalizations for psychotic disorders may be due to the increasing cannabis consumption in the Portuguese population, particularly influenced by moderate/high dosage cannabis consumers.^35^

## Legalization of non-medical cannabis use

Cannabis legalization is not a binary option opposing commercial legalization to continuing prohibition, as it includes a variety of possible programs.^20-25,36,37^

Several countries and states have abandoned the prohibitionist approach to drug use, and different regulation models have been adopted: Belgium,^20,38^ the Netherlands,^20,22-24,37,39,40^ Spain,^23,24,37-39^ Uruguay,^22-24,38,41^ Canada,^24,42^ and, in the United States, 11 states and the District of Columbia.^20,22-25,36,43,44^

### *De facto* legalization

However, cannabis use for non-medical purposes has not yet been legalized in any European Union country. The Dutch coffee-shops and the Belgian and Spanish social/cannabis clubs represent models of cannabis control, intermediate situations between prohibition and “complete” legalization,^20,24,37,38^ considered a *de facto* legalization – a prohibition with an expediency principle.^20,37^

The Dutch policy dates back to 1976, when the Netherlands adopted a formal policy of non-enforcement for violations involving possession or sale up to 30 gram/person of cannabis, further reduced to 5 gram.^39,40^ The government does not control production, packaging, or price, nor can it legally tax cannabis products; on the other hand, it has the power to ban promotion.^23^ The coffee shop model has a set of rules intended to control and limit cannabis trade and use.^40^ Additionally, from 2011, coffee shops started to be run as private clubs for Dutch citizens, excluding foreign citizens.^40^

Some considerations must be made about the Dutch experience, both positive and negative.^40^ Dutch citizens consume cannabis at more modest rates than some of their European counterparts, and do not seem likely to escalate their use relative to the rest of Europe or United States.^40^ Some data suggest that this separation between soft and hard drug markets possibly reduced the access to hard drugs.^20,40^ While some argue that depenalization did not lead, in itself, to rises in population levels of cannabis use among adults nor young people,^20^ inferred evidence indicates that the Dutch retail system increased consumption, mainly in its early years (when it was open to 16-year-olds and there was more advertising than nowadays).^40^

In Spain, possession and use in a public place is subject to administrative sanctions or fines.^20^ However, people are allowed to grow their own cannabis, even though they cannot run private profiting cannabis enterprises. The first cannabis social club opened in Barcelona in 2001.^20^ These clubs are non-profit and must follow recommendations regarding limits on monthly personal amounts of cannabis, hours of operation, and membership; promotion is banned.^20^ Questions have been raised about this model since legal cannabis access is limited to people who grow it for personal use or are invited into a social club.^23^ Therefore, some people may be forced to rely on black market sources.^23^

### Legalization and regulation

Cannabis has been legalized and regulated for non-medical use in Uruguay,^22-24,38,41,45^ Canada,^24,42,45^ and some U.S. states and the district of Columbia.^20,22-25,36,43-45^ Nations like Colombia, Luxembourg, Mexico, the Netherlands, New Zealand, and Switzerland are currently discussing cannabis legalization.^46^

#### Uruguay

Uruguay became, in December 2013, the first country in the world to legalize cannabis production, supply and non-medical use by adults.^22,24,41^ Theoretically, there are three mutually exclusive methods by which nationals can obtain cannabis: self-cultivation, purchasing marijuana at pharmacies, or joining a cannabis club that produces cannabis for its members. Uruguayans must choose one legal way and register with the Uruguayan Instituto de Regulación y Control del Cannabis.^23,38,41^ Cultivation is limited per person (no more than six plants at home) and private clubs can be formed to increase production (cannabis clubs, where adult users cultivate cannabis collectively for their own consumption with non-profit aims).^24^ Advantages of the cannabis clubs include control of product quality, job creation, and reduced risky consumption; on the other hand, they could be used as a cover for profit-driven businesses.^38^ Cannabis sale is controlled by the government through a network of licensed sale points and by establishing the retail selling price.^23,24^ Smoking at the workplace and driving vehicles under the effect of cannabis is illegal; the legal consequences include fines, destruction of stored cannabis, and being prevented from legally buying cannabis.^24^ Uruguayan public authorities have produced educational documents that aim to educate the population about the risks of consuming cannabis,^47^ a line of intervention that appears to be working, since the perception of risk among users has been rising since 2014.^48^ The success of Uruguay’s policies has been said to face some challenges related with under-registration of medium to high frequency consumers.^41^ Two possible major reasons that could restrain registration numbers are the philosophical views about consumption and individual rights and the fear of absence of confidentiality when registering.^23,41^ Nonetheless, in 2018, 30% of consumers were members of the regulated market and 60.3% of these people were registered as frequent users.^49^

After cannabis legalization in Uruguay, a reduction of circa 17% in the number of crimes directly associated with illegal drugs was observed: approximately 200 fewer crimes per year in 2017 (vs. 2013) – a decrease thought to be related to fewer drug possession prosecutions as a consequence of the legalization policy.^50^ On the other hand, the policy was followed by an increase in homicides connected with territorial and trafficking disputes between dealers.^50^ While the illegal trafficking of cannabis did not vanish, Uruguayan authorities have estimated a fivefold reduction in their activities from 2014 to 2018.^49^ Considerations are merited regarding morbidity and mortality after the legalization policies: problematic use of cannabis had not increased as of 2018, remaining stable at 16% of all users since 2011; in 2017, cannabis accounted for 1.2% of the total hospital admissions due to substance use; and no deaths as a consequence of cannabis consumption were recorded from 2012 to 2018^49^ – however, a recent study suggested that the legalization of non-medical cannabis may be associated with increases in fatal motor vehicle crashes, particularly those involving light motor-vehicle drivers and in urban settings.^51^ Cannabis experimentation and frequent use have been rising in Uruguay since 2006; although the Uruguayan drug observatory (Observatorio Uruguayo de Drogas) considers that these increases are a tendency that is independent of the recent cannabis policies.^48^ Although experimentation has been increasing in the country, studies have found preliminary evidence that the non-commercial Uruguayan model may not lead to important increases in adolescent cannabis use in the short-term.^52^

#### United States

Oregon, Washington DC, and Colorado have commissions to control sellers’ activities and sales to people under the age of 21 are prohibited.^23^ Cannabis-impaired driving is banned.^23^ Products’ constituents and labels are state-controlled.^23^ Promotion is permitted in Washington and Colorado (except to youth under the age of 21),^23^ but not in Oregon.^24^ In Colorado, an 80% decrease in law-enforcement and judicial costs related to cannabis was observed between 2010 and 2014 and overall expenses related to drug combat have reduced by 23% since 2010.^24^ After the opening of cannabis stores in 2014, arrests associated with synthetic cannabis reduced significantly.^24^ The reduction in synthetic cannabis supply is described as one of the benefits of legalization.^24^ Moreover, a reduction in chronic pain admissions was observed in Colorado, which is a finding that has been consistently reported in the literature, for legalization of both non-medical and medicinal cannabis use.^43^

But legalization has not been a bed of roses in the United States. A large, nationally representative survey with 505,796 respondents comparing marijuana use before and after the legalization of non-medical marijuana in the United States (2008-2016) identified increases not only in marijuana use but also in cannabis use disorder.^44^ In Colorado specifically, cannabis legalization led to increased marijuana use amongst young adults (18-25-year-olds), when compared with non-legalized states; greater exposure of children less than 10 years old, mostly to infused edible products (e.g., cookies, candies, sodas); the average potency of cannabis flower has increased^36^ (which is associated with worse health outcomes^21,24,26,36,46^); there is more cannabis abuse in general,^43^ particularly among individuals aged 26 and over^44^; numbers of emergency department visits and hospitalizations with marijuana-related billing codes have risen following legalization^36,43,53^; a 46% increase in cyclic vomiting was reported between 2010 and 2014 in the Colorado State Inpatient Database^45^; emergency department presentations for mental illness with a cannabis-related code increased five times faster than mental illness presentations without such a code between 2012 and 2014 (the largest increases being for persons with schizophrenia and other psychotic disorders, suicide and intentional self-harm, and mood disorders)^45^; cannabis dispensary workers without medical training have been reported to give potentially harmful medical advice to patients (e.g., advising them to stop their usual medications and use the cannabis product instead); and legalization has been associated with increased rates of hospitalization for motor vehicle accidents,^43,54^ alcohol abuse, and overdose injury.^43,53^ The increase in cannabis potency (meaning a higher tetrahydrocannabinol: cannabidiol ratio) was also observed in Washington,^24^ and it is believed to be a consequence of investments in innovation, development and marketing of characteristic products with for-profit goals.^24,36^ This is the case because Washington’s and Colorado’s legal frameworks allow the price of cannabis to be set by the market itself.^24,36^ Although cannabis potency has been rising both in Europe and the United States, the increase has been more pronounced in the latter.^55^ There are some concerns regarding the possible rise of “Big Cannabis” and its associated lobbying and marketing influence,^23,46^ since states only have limited control over supply and price, and allow product promotion.^23^

#### Canada

On 17 October 2018, Canada legalized and regulated non-medical use and supply of cannabis,^42^ making it the second nation to do so, after Uruguay. The dual goals of this legalization were to reduce youth use and eliminate the illicit cannabis market.^42^

Use of cannabis for non-medical purposes became legal across the country under the Cannabis Act which “creates a legal and regulatory framework for controlling the production, distribution, sale and possession of cannabis in Canada.”^42,56^ Persons aged 18 or older can possess up to 30 grams of dried or equivalent non-dried form in public.^42^ Adults are also allowed to make cannabis-infused food and drinks.^42^ Each household is allowed to grow up to four cannabis plants from licensed seed or seedlings.^42^ Each province sets its own procedures for retail sales, and these vary as to ownership or retail outlets (by the provincial government or private enterprise), but all include an option for on-line sales.^42^

The economic impact of this policy has recently been studied.^57^ The analysis of cash circulation pointed to the expected move of the majority of cannabis users from the black to the official economy.^57^ Statistics Canada calculates that around one-quarter of the cannabis market remains illegal or even slightly less over time.^57^ Economics experts have publicly stated that the legalization of cannabis could over time reduce the size of the total underground economy by around 4 to 5%, possibly leading some users to buy less of other illegal drugs and that the legalization of cannabis is likely to have fuelled a much larger decline in the total size of the black economy.^58^ On the other hand, 6 months after legalization, there were 260 cannabis retail stores across Canada: 181 privately run stores, 55 government-run stores, and 24 stores in the hybrid retail system.^59^ Compared to jurisdictions with a government-run model, jurisdictions with a private/hybrid retail model had 49% (95% confidence interval: 10-200) more stores *per capita* and stores were located closer to schools (median 166.7 m).^59^ In both retail models, there was over twice the concentration of cannabis stores in neighborhoods in the lowest income quintile compared to the highest income quintile.^59^ Evaluating the sales outcome, an October 2019 report stated that total cannabis sales for the first 7 months of the year were modest, probably because of the limited number of retail operations in Ontario and Quebec, where some 23 million people reside; each province had only 25 outlets, as of October 2019.^60^ The relatively high cost of the legal product – almost double that of the black market – and the limited types of product (dried flowers and oils) also worked against retail sales.^60^ In late 2019, more stores were being opened and a wider range of legal cannabis was becoming available, including edibles and topical and vaping products.^60^

Importantly, several Canadian health authorities jointly developed the “Lower- Risk Cannabis Use Guidelines,”^61^ an evidence-based educational initiative with different and complementary applications, not only improving health workers’ psychoeducation, but also constituting a tool for consumers or their peers.

## Discussion

### Outcomes of non-medical cannabis legalization policies

Legalization of non-medical cannabis use has had more than a few generalizable benefits: significant decreases in cannabis-related crimes, law-enforcement and judicial costs; reduction in synthetic cannabis supply; decline in black economies; and tax revenue increases. Besides the above, several other arguments have been stated to support the decriminalization and/or legalization pathways. Cannabis use is common among young adults and less deleterious than other substances already available, like alcohol or tobacco; the criminalization of cannabis use is more injurious than the substance itself, since users can be subject to criminal charges for consuming it and they often disproportionately affect ethnic minorities; legalization can enable regulation of the content of cannabis products, therefore protecting consumers.^45^ Nevertheless, one must not forget that cannabis is a known injurious substance: physically and mentally harmful, over the short and long term, to the user and also to their offspring. Unsurprisingly, legalization of cannabis use has been associated with several drawbacks. Mixed outcomes were not restricted to Uruguay (e.g., stabilization of cannabis-related treatment admissions, but increase of cannabis-related accidents and hospitalizations), since the U.S. states also reported several issues, mostly health-related (e.g., cannabis use and cannabis use disorders rose amongst young adults, greater exposure of children less than 10 years old, and increased average cannabis potency). Moreover, both nations reported rises in cannabis experimentation. Box 1 presents a summary of the greatest benefits and drawbacks of the different types of legalization policies.

**Box 1:** Cannabis legalization policies: relative advantages and disadvantages

	Advantages	Disadvantages
**Uruguay** Non-profit, centralized regime	- Significant decreases in cannabis-related crimes, law-enforcement and judicial costs - Reduction in synthetic cannabis supply - Decline in black economies - Tax revenue increases - Product quality control, limiting potency increases - Lower risk of profit-driven seller behavior	- Rise in cannabis experimentation - Increase in cannabis-related motor vehicles crashes - Dependent on the number of individuals willing to register as “consumers” - Cannabis clubs as possible refuge for profit-driven businesses
**United States** Profit-driven commercial regime	- Significant decreases in cannabis-related crimes, law-enforcement and judicial costs - Reduction in synthetic cannabis supply - Decline in black economies - Tax revenue increases	- Rise in cannabis experimentation - Increases in cannabis use disorders, presentations of mental illness with a cannabis-related code and cannabis-related hospitalizations - Increase in cannabis-related motor vehicle crashes - Market-shaped price - Rise of “Big Cannabis”? - Increase in cannabis potency - Cannabis dispensary workers/companies targeting profit, instead of public health
Canada* Mixed regime	- Significant decreases in cannabis-related crimes, law-enforcement and judicial costs - Reduction in synthetic cannabis supply - Decline in black economies - Tax revenue increases	- High cost of legal product may drive consumers to the black market

* Newer policy, with limited data.

These elements outline the careful and thoughtful considerations that ought to be undertaken before legislating. One good example is the importance of regulation: as control over product formats, content, and price should be considered in order to avoid problems like those reported in Washington (increased potency driven by for-profit motives) or to adapt to issues comparable to Oregon’s – where studies have suggested that publicity restrictions are important in order to protect citizens (particularly young adults) against pro-cannabis messages.^24^ The profit-driven commercial marketing of cannabis poses significant dangers and cannabis retail should instead be regulated from a public health point of view – a matter recently discussed by Simon Lenton.^62^ Regulatory obligations in retails cannabis sales must be respected and, if not, the penalties for non-compliance should compel business owners to guarantee that their employees behave according to concepts of harm reduction, instead of engaging in careless selling aiming for the highest profit.^62^ In this respect, New Zealand seems to be a step ahead, as the draft bill for the referendum regarding non-medical legalization of cannabis already encompassed offences for consumption facilities and cannabis outlets for which the penalties were higher for managers than for other comparatively unimportant employees, with possible suspension of licenses.^63^ This draft bill meant that if the owners of cannabis businesses wished to profit, they would have been required to fulfill public health obligations.^62^ However, 50.7% did not support the proposed Cannabis Legalization and Control Bill in the referendum.^64^

Canada’s legal non-medical cannabis system needs to attain maturity before any accurate assessments can be made regarding, for example, inferences on changes in cannabis use and harms. It will also be interesting to see if the future matches economists’ predictions in regard to shrinking of the underground economy.

Hall et al.^46^ have summarized several existing strategies that help reduce cannabis-related harm after legalization – some of which have been clearly implemented in the examples stated above. Governments can:

- Create a monopoly on cannabis production and sales (e.g., Uruguay);
- Use taxes to discourage heavy use (e.g., Canada);
- Decrease the number and location of retail cannabis outlets;
- Restrict advertising and promotions (e.g., Oregon);
- Educate the population on how to minimize the harms of cannabis (e.g., Uruguay, Canada, and the United States);
- Discourage adolescents from initiating use (e.g., Uruguay, U.S. states in general, and Canada);
- Target vulnerable cannabis users for intervention and treat them (e.g., children and adolescents, and individuals diagnosed with schizophrenia);
- Discourage routes of administration reliant on combustion;
- Dissuade people from using cannabis while driving (e.g., Uruguay, U.S. states in general, and Canada).

Along the same lines, and in the aftermath of legalization in the United States, Pardo^65^ proposed that non-medical cannabis legalization policies should promote a public health perspective, avoiding early commercialization and focusing on non-commercial models, maintaining price, rigorously regulating potentially harmful formulations of the substance, and controlling production and supply, while ensuring public security.

Following Colorado and Washington’s cannabis policies, Kilmer^66^ published a paper mainly aimed at decision makers that described 14 important factors (“the 14 Ps”) to be considered when legislating the non-medical use of cannabis, illustrating the complexity and the numerous models that can be implemented, as follows: 1) production; 2) profit motive; 3) power to regulate; 4) promotion; 5) prevention and treatment; 6) policing and enforcement; 7) penalties; 8) prior criminal records; 9) product types; 10) potency; 11) purity; 12) price; 13) preferences for licenses; and 14) permanency.

Naturally, and given the inexistence of comprehensive data, such innovative and broad policies have to be conceived in a flexible way, since they will require continuous and frequent data collection, allowing for future adaptations when necessary, because their initial forms will surely – and understandably – lack perfection.^65^

Many ideas can be proposed and conclusions drawn from the data available at the present time, which may help to pave the way for the future. However, most of the true public health effects of cannabis legalization are still unknown, for we are still in the early stages of these policies and their implications.

### A generic proposal for non-medical cannabis legalization in Portugal

When discussing legalization of recreational cannabis use, it should be taken into consideration that regulation approaches must be adjusted/tailored to the particular region/country’s context, considering social, cultural, and economic aspects. Analysis of international strategies and their outcomes ought to be taken into consideration in the debate on non-medical cannabis legalization and in development of policies, whether in Portugal or elsewhere around the globe.

In accordance with our review, we propose general guidelines for national policies on legalization of non-medical cannabis use in Portugal. However, they are not exclusive, since they follow ideas that have the potential to be tailored to fit the context of each country.

A non-medical cannabis use legalization policy in Portugal would probably be most useful and beneficial if developed from a public health perspective, as a non-commercial centralized model in order to avoid the dangers of profit-driven models. We believe the following points constitute the baseline for the development of such a strategy:

- Centralized control of product price, production, supply, quality, and potency;
- Establishment of regulatory obligations in retail cannabis sales and penalties for non-compliance mainly aimed at business managers;
- Publicity restrictions to protect against pro-cannabis messages;
- Education of the population about use and risks of consuming cannabis;
- Identification and treatment of vulnerable users;
- Restriction of possession amounts and public use;
- Prohibition of working and driving while under the influence of the drug;
- Implementation of systematic evaluations of the policy, allowing for fast and flexible adaptations when required.

### Implications and future research

Our study helps summarize the theoretical background for development of more structured and prospective research on cannabis legalization, showing that we are still in the initial stages of such policies, as the predominant consequences and a comprehensive framework are only expected to be available in the years to come, when a greater number of countries are anticipated to follow the legalization path, and long-term data on these policies start to become available.

Our paper provides a generic, theoretical, and science-driven proposal of cannabis legalization that can be of assistance to implementation of such a policy in Portugal (but also possibly in other nations), if legalization is to be pursued.

Future studies should focus on systematic evaluation of the pros and cons of the legalization of cannabis worldwide. One must be aware that attempts to estimate the outcomes of a drug policy change are complicated by the numerous exogenous factors (e.g., the interaction with other policies and social, cultural, and economic aspects) that influence drug-related harms and drug use. This is why future research ought to be undertaken not purely from economic or social perspectives, but including public health angles and, particularly, the short, medium, and long-term impact of these policies on the mental health of populations (whether healthy or with a history of psychiatric disorders).

Specific structured studies are required to assess the time-evolving consequences of legalization with respect to several social, economic, and health factors: populations’ consumption habits (initiation of use, frequency, duration and rates of dependency); perception of the risk of use and attitude towards use; cannabis-related car crashes; cannabis-related healthcare use; criminal and judicial spending; tax-revenue; and the impact on mental disorders and, specifically, on the use of other drugs and on populations seeking help.

Such research can provide flexibility, enabling the necessary rapid adaptation of policies and development of prophylactic and therapeutic evidence-based interventions.

### Strengths

We undertook an up-to-date and comprehensive review of cannabis legalization policies and their short-to-medium-term outcomes, attempting to impartially and equitably report and compare them. Working from this overview, we developed a scientifically-driven perspective on how to adequately apply these learnings to the Portuguese context and, possibly, in other nations.

While identifying and summarizing theory about the aforementioned topic, our study helps reinforce the foundations for development of more structured and prospective research on cannabis legalization, while at the same time identifying the research path to be followed.

### Limitations

As a non-systematic literature review, our study is not free from biases such as authors’ assumptions, nor is it replicable. Being a narrative review, our study may not include all the appropriate literature. The existence of bias due to political and social agendas behind the gray literature included cannot be ruled out.

Some differences found between the outcomes of policies may be due to the study methodology or a lack of studies or reports, and not to advantages of one strategy over the other (e.g., exposure of young children to cannabis products in the United States vs. no reference to them in Uruguay).

## Conclusion

The legalization of non-medical cannabis issue is a hot political subject with as-yet uncertain public health outcomes and balancing its advantages and disadvantages is no easy task. Although consumed globally, cannabis has the potential to cause detrimental physical and mental effects, with short and long-term consequences. Cannabis legalization has brought benefits where applied. Nonetheless, it has also brought downsides. Countries should carefully evaluate whether and how to decriminalize or legalize non-medical cannabis use, adapting such change to their own circumstances. If legalizing, cautious and thoughtful planning is of extreme importance, as is evaluating the lessons learned in the parts of the world where such changes have occurred. Public health-driven, and not profit-driven models, seem to be yielding the most benefits regarding non-medical cannabis legalization. Existing harm-reduction strategies can guide policy makers and positively contribute to public health, if the path of legalization is to be followed. Healthy dialogues between legislators and science should be encouraged, always keeping in mind that there are more than a few legalization pathways and that each choice influences, in a particular way, social and health wellbeing, government revenue, and job creation. The true public health effects of cannabis legalization are yet to be revealed, for we are still in the early stages of these policies and their implications. Future studies should systematically address the social, economic, and health consequences of legalization policies, paving the way for future policy adjustments and development of harm mitigation and treatment strategies.
